# Declined plasma sfrp5 concentration in patients with type 2 diabetes and latent autoimmune diabetes in adults

**DOI:** 10.12669/pjms.313.6964

**Published:** 2015

**Authors:** Liqing Cheng, Dongmei Zhang, Bing Chen

**Affiliations:** 1 Liqing Cheng, Department of Endocrinology and Metabolism, Southwest Hospital, Third Military Medical University, Chongqing, China, 400038; 2 Dongmei Zhang, Department of Dermatology, Southwest Hospital, Third Military Medical University, Chongqing, China, 400038; 3 Bing Chen, Department of Endocrinology and Metabolism, Southwest Hospital, Third Military Medical University, Chongqing, China, 400038

**Keywords:** Diabetes, Secreted frizzled-related protein 5, Inflammation, Insulin resistance

## Abstract

**Objective:**

Secreted frizzled-related protein 5 (sfrp5), like adiponectin, has been identified as a novel insulin-sensitising and anti-inflammatory adipokine. Our objective was to determine whether differences of circulating plasma sfrp5 concentration exist among type 2 diabetes (T2D), latent autoimmune diabetes in adults (LADA) and healthy population.

**Methods:**

Enzyme-linked immuno sorbent assay was employed to detect the circulating sfrp5 level in plasma, and other lab tests such as fasting glucose and creatinine were also examined. Correlation analysis between sfrp5 and characteristics of subjects was conducted IBM SPSS Statistics and GraphPad Prism.

**Results:**

Circulating sfrp5 level was significantly decreased in T2D and LADA patients plasma compared with that in healthy control (14.14±11.91ng/mL, 14.82±11.27ng/mL, 22.98±12.36ng/mL, respectively), although no differences was observed between LADA and T2D groups. Furthermore, we found sfrp5 was correlated with homeostasis model assessment of insulin resistance (HOMA-IR), diabetes duration and BMI. Finally we found sfrp5 was still negatively correlated with HOMA-IR after being adjusted for disease duration and BMI(r= -0.315, P< 0.05).

**Conclusions:**

Our results support a role for SFRP5 as a protective factor in the pathogenesis of autoimmune diabetes and facilitate a novel aspect for diabetes research.

## INTRODUCTION

Clinical and molecular studies have offered clear evidence for the role of diet-induced chronic low-grade inflammation as an important link between obesity and type 2 diabetes (T2D) and a wealth of pro-inflammatory and anti-inflammatory cytokines were associated.^1^-^3^ Furthermore, there is even a debate on T2D as an autoimmune disease, a hypothesis both metabolic and adipocyte stress acting via pro-inflammatory cytokines and inflammatory signaling followed by T cells, B cells and macrophages activated.^4^,^5^ Latent autoimmune diabetes in adults (LADA), a condition that shares many features with type 1 diabetes and is often misdiagnosed as type 2 diabetes, is well known as a autoimmune disease,^6^ however it’s association with pro-inflammatory and anti-inflammatory cytokines is few and controversial.^7^-^9^

Researches have done a lot on pro-iflammatory and anti-inflammatory factors associated with diabetes, especially cytokines and adipokines secreted by adipose tissues and adipocytes.^10^,^11^ Secreted frizzled-related protein 5 (sfrp5), one of five secreted frizzled-related protein family members, is a novel anti-inflammation adipokine, which can restrain chronic inflammation and improve insulin sensitivity in obesity and diabetes and have been identified as one of the key adipocytokines of metabolic regulation and obesity-induced metabolic disorders.^10^,^12^ Studies show that all of sfrp family members have conserved cysteine-rich domain (CRD), which shares high homology with CRD of frizzled protein of wingless-type(Wnt) receptors. Thus sfrp proteins can negatively regulate Wnt pathway by competitive binding to Wnt ligands with frizzled protein receptors. Sfrp5 has been demonstrated to bind to the non-canonical Wnt molecules Wnt5a and Wnt11 to inhibit both canonical and non-canonical Wnt signaling in human tissue culture.^13^

There are several controversial studies on circulating sfrp5 concentration in obesity and type 2 diabetes patients^14^-^17^ as well as on animal experiments.^18^,^19^ However there is no such report including patients of LADA to our knowledge. In this study, we sought to determine whether differences of sfrp5 level exist in LADA, type 2 diabetes and healthy control populations.

## METHODS

### Human subjects

Fifty-eight type 2 diabetes patients, twenty-two LADA patients and forty healthy controls were enrolled into this study. All diabetic patients were clinically confirmed according to diagnostic criteria of WHO in 2006 or other where described.^6^,^20^ Exclusion criteria were: acute or chronic infectious or other immunological disease and cancer. Blood samples were collected after an overnight fast of 8 hours in the early morning from endocrinology department and health management center, southwest hospital, Chongqing, China. The Ethics Committee of the First Affiliated Hospital of the Third Military Medical University approved this study and each participant signed an informed consent.

### Clinical and lab test characteristics

Body weight, height and blood pressure were measured in all subjects according to standard protocols. Body mass index (BMI) was determined by the ratio of weight/height squared (kg/m2). Blood samples from all participants were collected in the early morning after 12h fasting. Plasma was stored in -80 after centrifuging. Fasting glucose (FPG), fasting insulin (FINS), total cholesterol (Tch), triglyceride (TG), high-density lipoprotein cholesterol (HDL-C), low-density lipoprotein cholesterol (LDL-C), uric acid (UA), urea nitrogen (UN), creatinine (Cr), and glycosylated hemoglobin A1c (HbA1c) were tested following the hospital routine. The homeostasis model assessment of insulin resistance (HOMA-IR) was determined by: fasting insulin (mIU/L) × fasting glucose (mmol/L)/22.5.^21^ As HOMA is a steady state model, only fasting glucose between 3.5-25.0 mmol/L and fasting insulin between 2.9 -28.7 mIU/L will be valid in this model.

Enzyme-linked immuno sorbent assay (Elisa) kit from Cusabio Life Science was employed to detect the plasma sfrp5 level according to the manufacture’s instruction. The plasma was diluted 4 hundredfold with diluting solution. The minimum detectable dose of human SFRP5 of this kit is typically less than 0.39 pg/ml. The coefficient of variation was <8% for intra-assay and that for Inter-assay was<10%.

### Statistical analysis

Analyses were performed by one-way ANOVA with IBM SPSS Statistics (version 19.0) and dot plots by GraphPad Prism (version 6.0). Data are given as mean ±SD. In all statistical tests, P values <0.05 were considered as significant. Correlations between Sfrp5 and other variables were assessed using Pearson correlation analyses or Spearman correlation analyses as needed. Partial correlation analysis was used to control diabetes duration and BMI.

## RESULTS

### Clinical and lab test characteristics

As shown in Table-I, all groups were age and gender matched. BMI, SBP, FPG, FINS, HOMA-IR, Tch and HDL-C were increased in the type 2 diabetes population compared with those of healthy controls; BMI, FPG, FINS, Cr and HDL-C were increased in the LADA population compared with those of healthy controls; Furthermore, type 2 diabetes group exhibited a significantly higher diabetes duration, BMI, SBP, FINS, HOMA-IR, UN and Cr, respectively, than those of LADA population.

**Table-I:** Characteristics of subjects.

Characteristics	HC	T2D	LADA
Number	40	58	22
Sex (male), n (%)	21(52.5%)	29(50.0%)	11 (50.0%)
Age (years)	50.7±8.1	53.3±8.5	49.3±6.4
Diabetes duration (months)&	-	86.2±70.5	18.9±25.7
BMI(kg/m2) *#&	23.4±1.4	24.8±3.2	21.5±4.3
SBP(mmHg)*&	125.7±15.7	132.8±18.5	119.2±17.4
DBP(mmHg)	77.3±11.8	77.5±12.3	81.0±14.5
FPG(mmol/L) *#	5.4±0.4	7.6±3.4	11.4±8.0
FINS(mIU/L) *#&	10.8±2.0	14.0±4.7	5.1±3.1
HOMA-IR*&	2.7±0.6	4.4±1.4	2.9±1.5
UN(mmol/L) &	5.4±1.4	6.6±3.4	4.7±1.7
Cr(mmol/L) #&	72.3±15.7	90.1±72.9	68.3±76.6
UA(mmol/L)	307.7±76.1	309.5±112.4	290.5±113.0
Tch(mmolLl) *	4.9±1.0	4.5±1.8	4.3±1.2
TG(mmol/L)	1.2±0.5	2.0±3.4	2.1±2.3
HDL-C (mmol/L) *#	1.5±0.3	1.3±0.6	1.1±0.4
LDL- C(mmol/L)	2.5±0.6	2.6±1.1	2.3±0.4

All values are shown as actual numbers or mean± standard deviation. HC, healthy controls;T2D, type 2 diabetes; LADA, Latent autoimmune diabetes in adults; BMI, body mass index; SBP, systolic blood pressure; DBP, diastolic blood pressure; FPG, fasting plasma glucose; FINS, fasting insulin; HOMA-IR, homeostasis model assessment of insulin resistance index; Tch, total cholesterol; TG, triglyceride; HDL-C, high-density lipoprotein-cholesterol; LDL-C, low-density lipoprotein-cholesterol; UN, urea nitrogen; Cr, creatinine; UA, uric acid.

* P <0.05 between HC and T2D,

# P <0.05 between HC and LADA,

& P <0.05 between T2D and LADA.

### Plasma sfrp5 concentration are decreased both in type 2 diabetes and LADA subjects

**Fig.1:**
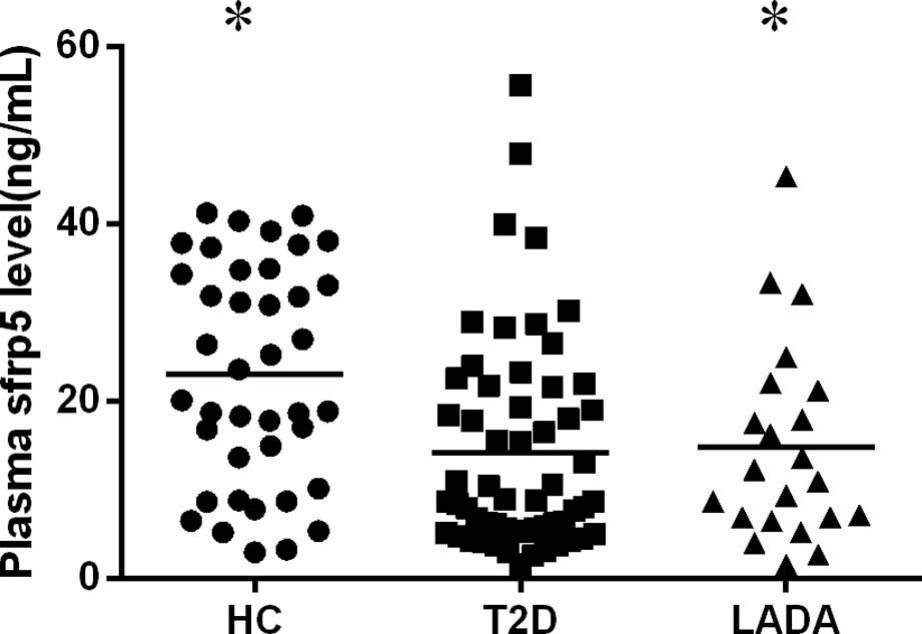
Plasma concentrations of sfrp5 in type 2 diabetes and LADA subjects determined by Elisa. HC, healthy controls; T2D, type 2 diabetes; LADA, Latent autoimmune diabetes in adults; * indicates significantly altered compared with HC group (P< 0.05).

We then measured sfrp5 protein concentrations in plasma of all subjects. In accordance with major results, patients with type 2 diabetes had declined plasma sfrp5compared with health control population (14.14±11.91ng/mL, 22.98±12.36ng/mL, respectively).^15^,^17^ As far as we know, plasma sfrp5 has not been determined in LADA population before. Here we found that plasma sfrp5 of LADA subjects was also significantly decreased (14.82±11.27ng/mL), although no lower than that of type 2 diabetes patients (Fig.1).

### Relationships between plasma sfrp5 concentrations and clinical and lab test values

We also analyzed the relationships between plasma sfrp5 level and clinical and lab test values with bivariate correlation analysis. We found a negative correlation between plasma sfrp5 level and several parameters, including diabetes duration, UA, HOMA-IR and BMI (r= -0.384,-0.239, -0.398, and-0.231, respectively, P< 0.05).

Furthermore, we sought to determine whether the correlation between sfrp5 and HOMA-IR were caused by disease duration and BMI, finally we still found sfrp5 was negatively correlated with HOMA-IR after being adjusted for disease duration and BMI(r= -0.315, p< 0.05).

## DISCUSSION

In the present study, we exhibited declined plasma sfrp5 level in T2D and LADA patients but with no differences between them. Furthermore, plasma sfrp5 was correlated with HOMA-IR, UA, diabetes duration, and BMI; especially the correlation between plasma sfrp5 and HOMA-IR still existed after adjustment for diabetes duration and BMI.

Sfrp5 is the fifth member of sfrp family, which has been observed to suppress Wnt5a signaling in adipose tissue.^18^ However, actually controversial behaviors of sfrp5 were reported. In sfrp5-mutated mice model fed a high-calorie diet, Ouchi et al.^18^ observed severe glucose intolerance and hepatic steatosis via activation of the c-Jun N-terminal kinase signaling pathway, while Mori et al. reported obesity-resistance via enhanced mitochondrial activities.^19^ In human beings, plasma sfrp5 concentration in T2D patients was declined in most reports.^14^,^15^,^17^ including ours, except one elevated.^16^ We figured that disagreement probably resulted from subjects with different diabetic durations and hence different inflammatory stages. The low sfrp5 level in LADA population somehow also can be explained by less secretion due to less fat and more consumption due to inflammatory status. The correlation with HOMA-IR, an extensively used model for insulin resistance, and BMI is not unexpected and well interpreted elsewhere,^18^ whereas the negative correlation with diabetes duration and UA is something worth thinking about. Probably with diabetes progressing and inflammation continuing, sfrp5 secreted is gradually consumed by pro-inflammatory Wnt pathway and finally exhausted. As to UA, A recent study indicates that it was associated with low-grade inflammation;^22^ It may also be a subsequence of diabetic renal damage.

It is well recognized that many major metabolic disorders, such as diabetes, results from obesity, which is associated with insulin resistance and a pro-inflammatory state.^23^ Expanded white adipose tissue (WAT) of obese subjects secretes many hormone (adipokine or adipocytokine), including sfrp5, leptin, adiponectin, TNF-α, resistin and IL-6, as critical players in regulating systemic lipid and glucose homeostasis as well as crosstalk between adipose tissue and other key metabolic organs, including the liver, muscle, and pancreas. Thus abnormal adipokine secretion and signaling often cause metabolic inflammation and disorders.^24^,^25^ Therefore, diabetes may results from multiple etiological factor and their interactions besides sfrp5 and more complicated underlying network remains to be elucidated.

As far as we know, there has been no such report simultaneously describing the circulating plasma sfrp5 concentration in healthy, LADA and T2D populations. These data demonstrate that sfrp5 may play a defensive role both in acute and low-grade chronic inflammation induced diabetes. Actually, sfrp5 expression is often down regulated in T2D patients, which may cause uncontrolled activation Wnt5a signaling. Therefore, Wnt5a overproduction and sfrp5 deficiency in diabetes may together play an important role in diabetes initiation.
